# Cardiomyopathy, familial dilated

**DOI:** 10.1186/1750-1172-1-27

**Published:** 2006-07-13

**Authors:** Matthew RG Taylor, Elisa Carniel, Luisa Mestroni

**Affiliations:** 1 University of Colorado Cardiovascular Institute and Adult Medical Genetics Program, Department of Internal Medicine, University of Colorado Health Sciences Center, Denver, Colorado, USA

## Abstract

Dilated cardiomyopathy (DCM) is a heart muscle disease characterized by ventricular dilatation and impaired systolic function. Patients with DCM suffer from heart failure, arrhythmia, and are at risk of premature death. DCM has a prevalence of one case out of 2500 individuals with an incidence of 7/100,000/year (but may be under diagnosed). In many cases the disease is inherited and is termed *familial *DCM (FDC). FDC may account for 20–48% of DCM. FDC is principally caused by genetic mutations in FDC genes that encode for cytoskeletal and sarcomeric proteins in the cardiac myocyte. Family history analysis is an important tool for identifying families affected by FDC. Standard criteria for evaluating FDC families have been published and the use of such criteria is increasing. Clinical genetic testing has been developed for some FDC genes and will be increasingly utilized for evaluating FDC families. Through the use of family screening by pedigree analysis and/or genetic testing, it is possible to identify patients at earlier, or even presymptomatic stages of their disease. This presents an opportunity to invoke lifestyle changes and to provide pharmacological therapy earlier in the course of disease. Genetic counseling is used to identify additional asymptomatic family members who are at risk of developing symptoms, allowing for regular screening of these individuals. The management of FDC focuses on limiting the progression of heart failure and controlling arrhythmia, and is based on currently accepted treatment guidelines for DCM. It includes general measures (salt and fluid restriction, treatment of hypertension, limitation of alcohol intake, control of body weight, moderate exercise) and pharmacotherapy. Cardiac resynchronization, implantable cardioverter defibrillators and left ventricular assist devices have progressively expanding usage. Patients with severe heart failure, severe reduction of the functional capacity and depressed left ventricular ejection fraction have a low survival rate and may require heart transplant.

## Background

Dilated cardiomyopathy (DCM) is a disease of the heart muscle characterized by ventricular dilatation and impaired systolic function [1]. DCM is a leading cause of heart failure and arrhythmia. Due to its significant prevalence, high mortality and morbidity, including frequent hospitalizations, DCM is a major health concern for adults. Despite improvements in the treatment of heart failure introduced in the last 10 years, including the general availability of cardiac transplantation and better medical treatment, clinical outcome following the onset of symptoms has not substantially changed. Mortality remains high, the disease is progressive and unrelenting, and disability and morbidity are among the highest of any disease or disease syndrome.

DCM is defined as *idiopathic *, when the disease appears sporadic, isolated in a single member of a family and without known cause, or *familial *when occurring in two or more related family members [1,2].

## Definition of DCM, diagnostic criteria of familial DCM and differential diagnosis

The diagnosis of DCM is made according to criteria provided by the World Health Organization/International Society and Federation of Cardiology (WHO/ISFC) [1], the Guidelines of the National Heart, Lung, and Blood Institute Workshop on the Prevalence and the Etiology of Idiopathic Dilated Cardiomyopathy [3] and the more recent Guidelines for the Study of Familial Dilated Cardiomyopathies [2], designed to improve the sensitivity and specificity of the old classification criteria.

DCM is defined by the presence of: a) fractional shortening (FS) less than 25% (> 2SD) and/or ejection fraction less than 45% (> 2SD); and b) left ventricular end diastolic diameter (LVEDD) greater than 117% (>2SD of the predicted value of 112% corrected for age and body surface area, BSA) [4], excluding any known cause of myocardial disease. In the context of a familial DCM, these criteria are used to diagnose the proband in a family.

A familial DCM (FDC) is defined by the presence of: a) two or more affected relatives with DCM meeting the above criteria; or b) a relative of a DCM patient with unexplained sudden death before the age of 35 years [2]. In FDC, family members may be classified as *affected *, *unaffected *or *unknown *[2]. This classification is based on *major *and *minor *criteria that have been developed to account for the high frequency of minor cardiac abnormalities within families with FDC and the need of more sensitive criteria [5,6].

In relatives, the *affected *status is defined by the presence of: a) 2 **major **criteria consisting of left ventricular systolic dysfunction (fractional shortening < 25% and/or ejection fraction < 45%) *and *dilatation (LVEDD > 117% of the predicted value corrected for age and BSA) [4] or b) left ventricular dilatation (as defined above) *and *one minor criterion; or c) 3 minor criteria. The *unknown *status is defined by the presence of 1 or 2 minor criteria and the *unaffected *status is defined by the presence of a normal heart or the determination of other causes of myocardial dysfunction.

**Minor **criteria of disease are: a) unexplained supraventricular arrhythmias (atrial fibrillation or sustained arrhythmias), or frequent (> 1000/24 h) or repetitive (3 or more ectopic beats with a heart rate > 120 beats/min) ventricular arrhythmias before the age of 50; b) left ventricular dilatation (> 112% of the predicted value); c) left ventricular dysfunction (ejection fraction < 50% or fractional shortening < 28%); d) unexplained cardiac conduction system disease (grade II or III atrio-ventricular blocks, complete left-ventricular bundle branch block, or sinus nodal dysfunction); e) unexplained sudden death or stroke before 50 years of age; f) segmental wall motion abnormalities (> 1 segment, or 1 if not previously present) in the absence of intraventricular conduction defect or ischemic heart disease.

**Exclusion **criteria for *idiopathic/familial *DCM are: a) blood pressure more than 160/110 mmHg, documented and confirmed through repeated measurements; b) obstruction (more than 50%) of a major branch of the coronary artery; c) alcohol intake more than 100 g/day; d) persistent high rate supraventricular arrhythmia; e) systemic diseases; f) pericardial diseases; g) congenital heart diseases; h) cor pulmonale; j) myocarditis.

DCM can be distinguished from other forms of secondary dilatation and dysfunction of the ventricles due to known cardiac or systemic processes [1]. These are referred to as secondary cardiomyopathies, named for the disease process with which they are associated, such as ischemic cardiomyopathy, valvular heart cardiomyopathy, hypertensive cardiomyopathy, alcoholic cardiomyopathy, and myocarditis. Less common forms of secondary cardiomyopathies are peripartum cardiomyopathy and cardiomyopathies developing in the setting of amyloidosis, hemochromatosis, sarcoidosis, and due to toxicity from agents like doxorubicin. In the pediatric population, metabolic cardiomyopathies are encountered with greater frequency. It should be noted that cases of myocarditis and peripartum cardiomyopathy can occur in a familial setting, where cases of DCM can also be present, and therefore, it may be difficult to classify these forms [5].

Table 1 shows the diagnostic tools available for differentiating the different forms of DCM.

**Table 1:** Etiology and diagnostic tools in Dilated cardiomyopathy (DCM)

**Causes**	**% of cases**	**Diagnostic tools**
***Frequent ***		
Idiopathic/Familial DCM	20 – 50	family history, echocardiogram, detailed evaluation of first degree relatives, coronary angiography, endomyocardial biopsy
Ischemic DCM	50 – 70	history, coronary angiography
Valvular DCM	1.5 – 4	echocardiogram, physical exam
Hypertensive DCM	2 – 4	physical exam, echocardiogram showing hypertrophy
Alcoholic	3 – 40	history of excessive alcohol use
Myocarditis	5 – 10	history compatible with viral myocarditis, endomyocardial biopsy
***Rare ***	2–3	
Peripartum		history
Amyloidosis		echocardiogram, endomyocardial biopsy, rectal/fat pad biopsy
Hemochromatosis		extra-cardiac signs, endomyocardial biopsy, iron studies
Sarcoidosis		extra-cardiac signs, endomyocardial biopsy
Doxorubicin toxicity		history of exposure to doxorubicin
Other toxic substances		history
Metabolic DCM		laboratory tests, pediatric age

## Epidemiology

DCM has a prevalence of one case out of 2500 individuals [7] with an incidence of 7/100,000/year [8]. However, DCM is probably under diagnosed and is now believed to account for a much larger number of cases, owing to the fact that subjects may remain asymptomatic until marked ventricular dysfunction has occurred.

Prior to 1990, FDC was not widely recognized and genetic contributions to the development of dilated cardiomyopathy were rarely implicated in disease models. In 1981, a retrospective review of 104 patients with DCM at the Mayo clinic estimated that approximately 2% of cases were potentially familial in nature [9]. The paradigm changed dramatically in 1992 when it was reported that by carefully collecting the family history and through the screening of relatives by physical examination and echocardiography, 20% of cases of DCM were likely familial [6]. More recent studies support that FDC may account for between 35% and 48% of seemingly *familial *DCM [10-13]. Unfortunately, there are no reliable clinical or morphologic parameters able to predict the familial form from non-genetic causes of cardiac dilatation. A result of this circumstance is that family history data has become critical to the evaluation of these patients and families.

## Clinical description

Patients initially present with signs and symptoms of heart failure, due either to volume overload or to low cardiac output. Usually, by the time of the diagnosis, probands (here referring to the first individual diagnosed within a family) have severe impairment of the left ventricular ejection function and are in New York Heart Association (NYHA) functional class III-IV. Affected relatives, on the other hand, can be asymptomatic with mild ventricular dilatation and dysfunction. Twenty to thirty-five percent of patients present with chest pain, mostly during exercise, and the electrocardiogram (ECG) may show pseudo-infarction Q waves. Angina pectoris is thought to be due to limited coronary vascular reserve. Fatigue is present in almost one third of patients. Palpitations are very common, due to ventricular arrhythmias, nevertheless, syncope and sudden death rarely constitute the first symptom of the disease. Pulmonary and systemic thromboembolisms occur, as first manifestation of the disease, at a rate of 1 to 6% per year. Most of them can be found in cases with severe left ventricular dilatation and dysfunction. A particular form of familial DCM due to mutation of the lamin A/C gene [14,15] presents with mild dilatation and severe dysfunction of the left ventricle, conduction defects, supraventricular arrhythmias, variable skeletal muscle involvement and variable serum creatine kinase (CPK) levels. The prognosis for many of these patients is not favorable. X-linked FDC, due to mutations in the dystrophin gene, has a less severe prognosis and presents with increased CPK, muscular abnormalities, and the typical signs of *dystrophinopathy *at the skeletal muscle biopsy [16-19].

## Diagnostic methods

The basic evaluation in the proband [2] consists of an accurate family history, physical examination with specific attention to the neuromuscular apparatus, laboratory examination including CPK, chest X-ray, ECG and echocardiogram. In selected cases, an exercise stress test or a pharmacological test, such as dobutamine echocardiography, may be indicated to induce ischemia and unmask an ischemic cardiomyopathy. More specific diagnostic tests include hemodynamic and coronary angiographic study, radionuclide ventriculography and endomyocardial biopsy. In the presence of neuromuscular abnormalities, needle skeletal muscle biopsy is indicated. Molecular genetic diagnosis should be performed when the test is available and may impact the clinical management. At present, molecular genetic analysis is available for the following FDC genes: *DES, DMD, LMNA, MYBPC3, MYH7, TAZ, TNNT2*, and *TMP1 *.

## Etiology

In FDC, there is clear evidence of Mendelian segregation of disease phenotype. FDC is a heterogeneous entity (Table 2). Different forms have been identified and should be distinguished based on patterns of transmission and characteristics of the phenotype. When classifiable, the clinical patterns encountered include: autosomal dominant FDC without extracardiac manifestations; autosomal recessive FDC; FDC with X-linked transmission; autosomal dominant FDC with subclinical skeletal muscle involvement; autosomal dominant FDC with conduction defects; autosomal dominant left ventricular non-compaction; unclassifiable FDC with retinitis pigmentosa and hearing loss [5,13]. Right ventricular cardiomyopathy/or arrhythmogenic right ventricular dysplasia is considered a distinct entity [1]. There is clear evidence of incomplete penetrance as well as age-dependent penetrance, exemplified by the fact that FDC is typically an adult-onset disease and many persons carrying mutations do not develop overt disease until their fifth or sixth decade of life [20].

**Table 2:** Known Familial Dilated Cardiomyopathy (FDC) genes and their OMIM references

**Phenotype**	**Frequency (%)**	**Chromosomal location**	**Locus**	**OMIM [40]**	**Gene symbol**	**Gene**
Autosomal dominant FDC	56	1q32	CMD1D	191045	*TNNT2 *	Cardiac troponin T
		3p21.1		191040	*TNNC1 *	Cardiac troponin C
		2q31	CMD1G	188840	*TTN *	Titin
		2q35	CMD1I	125660	*DES *	Desmin
		6q12-q16	CMD1K	172405	*PLN *	Phospholamban
		9	CMD1B	600884		
		10q21-q23	CMD1C	193065	*VCL *	Metavinculin
		11p11		600958	*MYBPC3 *	Myosin-binding protein C
		11p15.1	CMD1M	600824	*CSRP3 *	Cysteine-glycine-rich protein 3
		14q11.2-13	CMD1A	160760	*MYH7 *	Cardiac β-myosin heavy chain
		15q14	CMD1A	102540	*ACTC *	Cardiac actin
		15q22.1		191010	*TPM1 *	**α **-tropomyosin
		17q12	CMD1N	604488	*TCAP *	Tinin-cap (teletonin)
		10q23.2		605906	*LDB3 *	Cypher/ZASP
		12p12.1		601439	*ABCC9 *	Regulatory SUR2A subunit of cardiac K_ATP _channel

Autosomal recessive FDC	16	19q13.42		191044	TNNI3	Cardiac troponin I
		unknown		212110		

X-linked DCM	10	Xp21	XLCM	300377	*DMD *	Dystrophin

Autosomal dominant FDC	7.7	1q11-q23	LGMD1B	150330	*LMNA *	Lamin A/C
with skeletal muscle disease		5q33-34	LGMD2F	601411	*SGCD *	δ-sarcoglycan
		4q11	LGMD2E	600900	*SGCB *	β-sarcoglycan
		6q23	CMD1F	602067		

Autosomal dominant FDC	2.6	1q1-q1	CMD1A	150330	*LMNA *	Lamin A/C
with conduction defects		2q14-q22	CMD1H	604288		
		3p22.2	CMD1E	600163	*SCN5A *	Na channel, voltage-gated, type V, α polypeptide

RareFDC:	7.7					
-Left ventricular non-compaction		Xq28		300069	*TAZ *	G4.5 (tafazzin)
		18q12.1-q12.2		601239	*DTNA *	**α **-dystrobrevin
		10q23.2		605906	*LDB3 *	Cypher/ZASP
-Autosomal recessive with retinitis pigmentosa and deafness		6q23-q24	CMD1J	605362	*EYA4 *	Transcriptional coactivator EYA4
-Autosomal recessive with wooly hair and keratoderma		6p24		125647	*DSP *	Desmoplakin
X-linked congenital DCM		Xq28		300069	*TAZ *	G4.5 (tafazzin)

Mitochondrial DCM		mtDNA		510000		

Carefully designed studies of larger FDC families (by genetic linkage analysis and other methods) have implicated 29 chromosomal loci as containing FDC genes. Several different genes at these loci have been identified to date (Table 2). The majority of these genes encode proteins that have cytoskeletal and/or contractile properties. However, genes of the nucleoskeleton and, more recently, ion channel encoding genes are also relevant in FDC patients [15,21,22]. Most mutations described so far have been private mutations and are not shared between unrelated families. To date, studies have primarily focused on highly selected families and/or on relatively small numbers of families. Consequently, the genetic epidemiology of mutations in FDC genes across all FDC families remains unknown. Furthermore, as mutations have now been described in seemingly non-familial cardiomyopathies (*sporadic or truly "idiopathic" DCM *), the contribution of FDC gene mutations to isolated or *sporadic *dilated cardiomyopathies is not well-defined.

## Management and treatment of familial dilated cardiomyopathy

The management of FDC focuses on limiting the progression of heart failure and controlling arrhythmia.

### General measures

They include patient education, salt and fluid restriction, treatment of hypertension, limitation of alcohol intake, control of body weight, and encouraging moderate exercise, preferably aerobic in a controlled environment.

### Pharmacological therapy

Pharmacotherapy includes a multitude of agents [23] enclosed in the standard approaches to heart failure. Only few of them in each class will be mentioned here.

• *Angiotensin converting enzyme (ACE) inhibitors *reduce mortality, hospitalization and progression of heart failure, as noted in several trials including CONSENSUS [24], SOLVD [25], and SAVE [26]. These drugs are generally started at low doses and are gradually titrated up to doses equivalent to those showing efficacy in randomized trials. Captopril is increased to a maximum of 50 mg three times/day. The maximum dose of Enalapril is 20 mg twice/day and that for Lisinopril is 40 mg once/day. The highest tolerated doses provide the most benefit.

• *Angiotensin receptor blockers (ARBs) *provide a reasonable alternative to ACE inhibitors (in patients who are intolerant to these agents). The use of ARB is based on trials such as ELITE-I and II [27], Val-HeFT [28], and OPTIMAAL [29]. The dose of Losartan used in these trials was around 50 mg once/day. The maximum dose of Valsartan is 160 mg twice/day. The addition of an ARB to an ACE inhibitor likely offers little additional benefit based on the Val-HeFT trial.

• *First generation calcium channel blockers *are not recommended in heart failure patients.

• Trials using *endothelin antagonists *have been disappointing to date and these agents are not recommend in standard guidelines [23].

• *Diuretics *have not been assessed in a randomized study to verify their effect on survival in heart failure. Furosemide is used at daily doses of 20 mg to 600 mg daily. Bemetanide and ethacrynic acid are other loop diuretics currently in use. Torsemide has better bioavailability when taken orally and is used in some patients who do not respond to oral furosemide. Frequently, the dose is half of that of furosemide for a similar effect. When high doses of loop diuretics are needed and, especially if the patient has diuretic resistance, metolazone is added, usually at a dose of 2.5 mg once/day to 5 mg twice/day. Acetazolamide is used at a dose of 250 to 500 mg daily in some patients with metabolic alkalosis.

• *Aldosterone inhibitors *such as spironolactone and eplerenone reduce mortality, as noted in RALES [30] and EPHESUS [31], respectively. Spironolactone is used at doses varying from 12.5 to 50 mg once/day. Eplerenone was started with a dose of 25 mg orally once/day, which was increased to 50 mg once/day as tolerated. Eplerenone is more selective for the aldosterone receptor and avoids some of the side-effects associated with spironolactone, such as gynecomastia.

• *Vasopressin antagonists *are still under investigation.

• *Natriuretic peptides *are available only intravenously for acute decompensation.

• The only *inotrope*, which use is widely accepted in chronic heart failure, is digoxin. It is dosed at 0.125–0.25 mg once/day and is adjusted to renal function. A recent post-hoc analysis of the Digitalis Investogators Group [32] divided the patients based on their digoxin serum level and showed that all-cause mortality was reduced by 6.3% in the subgroup of patients whose level was between 0.5 and 0.8 ng/ml.

• *Beta-adrenergic blockers *are considered a major advance in the therapy of heart failure. CIBIS-II [33] and MERIT-HF [34] showed a 34% relative reduction in all-cause mortality using bisoprolol and metoprolol succinate (both beta-1 selective blockers). Carvedilol, a non-selective beta-blocker with alpha-blocking properties reduced mortality by 35% in severe heart failure (COPERNICUS) [35]. Beta-blocking agents must be titrated gradually towards their target doses. The target dose of carvedilol is 25 mg twice/day in patients less than 70 kg, and 50 mg twice/day in heavier patients.

• The use of *anticoagulants/antiplatelet agents *is a controversial subject and more studies are underway to identify which therapy to use and in whom. In clinical practice, anticoagulation is often used in patients with a left ventricular ejection fraction less than 30%.

### Mechanical devices

The use of cardiac resynchronization therapy (CRT) with biventricular pacing improves symptoms in advanced heart failure and reduces hospitalizations and mortality. In the COMPANION trial [36] CRT reduced the risk of death and hospitalization for any cause by approximately 20%, with a 50% reduction in the risk of death for any cause in the subgroup of patients with non-ischemic DCM randomized to receive CRT + defibrillator. In the treatment of arrhythmias, the use of implantable cardioverter defibrillators (ICDs) is being progressively expanded as they are proving to be useful in reducing mortality from sudden death compared to antiarrhythmic drugs. The recently published SCD-HeFT trial [37] showed a 23% relative risk reduction of the primary endpoint of death from any cause in the ICD arm compared with placebo. The use of left ventricular assist devices (LVAD) was evaluated in REMATCH [38]. All-cause mortality was 52% at one year in the LVAD group versus 25% in the medical group. The benefits of LVADs are tempered by a multitude of device-related complications.

Finally, patients with severe heart failure, severe reduction of the functional capacity and depressed left ventricular ejection fraction have a low survival rate and may require heart transplant. In this setting, heart transplantation improves survival and quality of life.

### Asymptomatic left ventricular dysfunction

So far, no trials exist on the usefulness of therapy in asymptomatic affected relatives. However, based on studies on ischemic heart disease, it is believed that an early use of ACE inhibitors and/or beta adrenergic blockers could be very important in slowing the progression of the disease.

## Genetic counseling

Genetic counseling approaches for familial FDC are still under-developed and consensus guidelines are lacking. The high degree of genetic heterogeneity, uncertainty as to which familial DCM genes are commonly mutated, and the propensity to uncover private mutations, have combined to hinder the development and widespread application of clinical testing. As the disease is of relatively late onset, many individuals in older generations may be unavailable for examination or may be deceased. Limited knowledge of FDC and thorough evaluation of relatives at risk by practicing generalists and cardiologists likely also contributes to under-recognition of this condition.

An accurate family history and screening of first-degree relatives has fundamental importance in FDC. This approach is not yet widely practiced in the United States. Each first-degree relative should undergo a detailed physical examination, ECG, and echocardiogram. Serum creatinine kinase levels can also be useful, especially if the proband has elevation of this marker. Signal averaged electrocardiography (SAECG) has also been suggested as an additional diagnostic tool [39]. The thorough evaluation of relatives of patients is standard for the research studies of FDC.

For families where a pathogenetic mutation has been detected, the evaluation of relatives at risk can include molecular testing to confirm the presence or absence of the pathologic mutation. This information can be integrated into the genetic counseling provided to each individual within that family. Non-carriers may be reassured that they are not at increased risk of developing FDC. Asymptomatic carriers who are at increased risk of disease may be considered for regular evaluations (including echocardiogram) to screen for the development of early disease. As incomplete penetrance is a feature of this disease, the counseling session of asymptomatic mutation carriers should focus on the *increased risk *of developing FDC for mutation carriers, rather on models that implies a *certainty *of disease for all mutation carriers.

In families where a pathologic mutation has not been detected, genetic counseling is understandably less precise. A detailed examination and analysis of the pedigree is essential to define, if possible, the likely mode of inheritance. Implicit in this exercise is that historical information provided about relatives should be confirmed through either direct evaluation of relatives or reviewing medical records and/or autopsy reports. In performing the genetic counseling, careful attention to the problems of age-dependent and incomplete penetrance should be integrated into the discussion. Relatives judged to be at significantly increased risk of developing a cardiomyopathy should be considered, as above, for regular screening.

## Antenatal diagnosis

The use of molecular genetic testing to test fetuses for adult-onset disease has been the subject of a number of ethical discussions. In spite of initial widespread reluctance to embark upon testing in this setting, a number of recent examples are challenging this paradigm. Such discussions are likely to ensue in the context of familial DCM once clinical genetic testing and counseling becomes more widely available.

## Unresolved questions

There are many aspects of familial DCM that remain poorly understood. A clear and comprehensive understanding of the genetic epidemiology of gene mutations in both FDC and sporadic DCM is needed. Such knowledge will provide a foundation for the development and continued improvement of genetic testing, which will likely be panel-based to simultaneously test multiple genes. Animal models of FDC have been developed to better characterize the pathogenesis of how the underlying genetic defects initiate and/or propagate the disease process. Ultimately, this work may suggest novel approaches to target the underlying molecular defects. There is evidence of substantial variability in phenotype within families, suggesting that environmental and/or modifying genetic factors may interact with the underlying FDC mutations. Pilot studies to explore these hypotheses are now underway. Finally, experimental approaches to explore how conventional cardiovascular therapies impact the disease process are desperately needed.
